# Soluble Production, Characterization, and Structural Aesthetics of an Industrially Important Thermostable *β*-Glucosidase from *Clostridium thermocellum* in *Escherichia coli*

**DOI:** 10.1155/2019/9308593

**Published:** 2019-11-07

**Authors:** Syed Shoaib Ahmed, Mohsina Akhter, Muhammad Sajjad, Roquyya Gul, Sana Khurshid

**Affiliations:** ^1^Institute of Molecular Biology and Biotechnology, Centre for Research in Molecular Medicine, The University of Lahore, Bhobatian Chowk, 1-Km Defence Road, Lahore 54500, Pakistan; ^2^School of Biological Sciences, Quaid-e-Azam Campus, University of the Punjab, Opp. Sheikh Zaid Hospital, Canal Bank Road, Lahore 54590, Pakistan; ^3^Department of Life Sciences, University of Management and Technology, Johar Town, Lahore 54000, Pakistan; ^4^Faculty of Life Sciences, Gulab Devi Educational Complex, Ferozpur Road, Lahore 54000, Pakistan

## Abstract

This study aims to achieve high-level soluble expression and characterization of a thermostable industrially important enzyme, i.e., beta-glucosidase (BglA; EC: 3.2.1.21), from *Clostridium thermocellum* (*C. thermocellum*) by cloning in an *Escherichia coli* (*E. coli*) expression system. BglA was expressed as a partially soluble component of total cellular protein (TCP) having a molecular weight of ∼53 kDa with 50% of it as soluble fraction. Purification in two steps, namely, heat inactivation and Ni-chromatography, yielded approximately 30% and 15% of BglA, respectively. The purified (∼98%) BglA enzyme showed promising activity against the salicin substrate having a *K*_m_ of 19.83 mM and a *V*_max_ of 0.12 *μ*mol/min. The enzyme had an optimal temperature and pH of 50°C and 7.0, respectively, while retaining its catalytic activity up till 60°C and at pH 7. The optimized maximum expression level was attained in M9NG medium with lactose as an inducer. Circular dichroism revealed presence of alpha helix (43.50%) and small percentage of beta sheets (10.60%). Factors like high-end cellulolytic activity, fair thermal stability, stability against low pH, and ease of purification make BglA from *C. thermocellum* a potential candidate in industrial applications.

## 1. Introduction

Cellulose is one of the polymers of glucose, having a headstrong lysis activity but is still renewable. This lytic reaction consists of endoglucanases and exoglucanases fundamental activities, by acting on cellulose, and produces cellobiose with cellooligosaccharides. At this stage, beta-glucosidase converts cellobiose and cellooligosaccharides into glucose molecules. Glucose produced in this instance imparts end product inhibition through stearic hindrance to the glucanases. Alternatively, the processive endoglucanases had their own mechanism for converting cellulose into glucose, which is equivalent to endoglucanases and cellobiohydrolases. Thus, utilization or removal of excessive cellobiose from the system by the activity of beta-glucosidase evades the inhibition caused by cellobiose accumulation. Therefore, glucosidase is a part of well-characterized cellulolytic systems along with other cellulases [1, 2].

*Clostridium thermocellum* (*C. thermocellum*) is a cellulolytic thermophile, which uses a highly active enzymatic structure having a collection of exoglucanases and endoglucanases along with two beta-glucosidases, namely, BglA and BglB. Its cellulolytic activity is, however, affected by the accumulation of cellobiose, mainly due to inhibition of beta-glucosidases. This inhibition if somehow catered will not only enhance the extent of final glucose conversion from cellobiose but can also elevate the reaction rate [3].

Most industrial processes make use of higher temperatures, as they enhance the catalysis rate as well as hydrolytic efficiency.  Table 1 shows a comparison of several tested beta-glucosidases indigenously produced by different microorganisms at industrial scale by the fermentation process.

Thus, search of thermostable enzymes which can withstand higher temperatures are active nowadays. Several cellulosic components like xylanases and endoglucanases of *C. thermocellum* have proven to be competent enough with respect to their higher catalytic activity and thermostability, which implies that their glucosidase activity may also be loftier [11, 12]. The present study was designed to clone and obtain soluble production of BglA from *C. thermocellum* in an *Escherichia coli* (*E. coli*) expression system with a better yield to be utilized in an industry at a cost-effective level. Furthermore, it was purified and characterized to attain its improved biological activity and thermostability.

## 2. Materials and Methods

### 2.1. Materials

In the current study, genomic DNA of *C. thermocellum* (ATCC® 27405D-5™) was procured from ATCC. The *E. coli* strains used for the propagation and expression of BglA were DH5 and BL21-CodonPlus (DE3)-RIPL, respectively. For cloning and expression, different vectors were utilized, i.e., pTZ57R/T (Fermentas Inc., Ontario, Canada) and pET-28a (+) (Novagen, Madison, USA), respectively. NcoI and XhoI (Fermentas Inc., Ontario, Canada) restriction enzymes were used for restriction of cloning and expression vectors. However, InsTAclone™, TransformAid™, DNA/plasmid extraction, and BigDye Terminator v3.1 Cycle Sequencing kits utilized in the present study were obtained from Thermo Scientific.

### 2.2. T/A Cloning and Sequencing of BglA

The *C. thermocellum* genomic DNA was used to amplify bglA gene-coding sequence by using a pair of primers with 5′ upstream restriction recognition sequences to integrate the restriction sites in an amplicon. The forward primer 5′GC*CCATGG*CAAAGATAACTTTCC3′ with NcoI (italicized) and reverse primer 5′GC*CTCGAG*AAAACCGTTGTTTTTGATTA3′ with XhoI (italicized) sites were used. The thermal cycling conditions were set as 5 min at 95°C, 30 cycles of 45 sec at 94°C, 45 sec at 58°C, and 1 min at 72°C with a final extension phase at 72°C for 20 minutes. The amplified bglA amplicon (1350 bp) was gel purified and ligated in a linear cloning vector, i.e., pTZ57R/T using InsTAclone™ kit and transformed into an *E. coli* strain, DH5*α*, via TransformAid™ kit according to the manufacturer's protocol.

The confirmed transformants were selectively propagated for sequence analysis of *bglA* gene from pTZ57R-*bglA* recombinant plasmid. The dideoxy chain termination method was used for sequencing using BigDye Terminator v3.1 Cycle Sequencing kit on the GA 3500 genetic analyzer (Applied Biosystems™).

### 2.3. Construction of Expression Vector

For the construction of expression vector, the recombinant plasmid (pTZ57R-bglA) was restriction digested with NcoI/XhoI enzymes to obtain the insert for pET-28a (+) vector. Prior to ligation, the vector was also digested with the same enzymes. The ligated vector (pET28a-bglA) was twice transformed into *E. coli* strains; first in cloning strain DH5*α*, later the positive plasmid was retransformed into expression strain BL21 CodonPlus (DE3)-RIPL. The positive transformants were confirmed by colony PCR and restriction analysis.

The confirmed transformants of *E. coli* BL21 CodonPlus (DE3)-RIPL cells were processed for expression of BglA enzyme. 50 ml Luria-Bertani (LB) medium supplemented with kanamycin (100 *μ*g/ml) was used for culturing overnight at 37°C and 150 rpm. Next morning, 100 ml LB was supplemented with 2% overnight culture and allowed to grow up to an OD_600_ of 0.8 at 37°C. At this point the cells were induced with 0.5 mM IPTG, OD_600_ was measured every 2 hours and cells were harvested as they reached stationary phase. To confirm the expression of bglA gene as BglA enzyme, cells of 1 ml culture were pelleted and resuspended in lysis buffer (1.5 M Tris-Cl, 0.5 mM PMSF). Cells were then lysed by sonication and centrifuged to separate the inclusion body and soluble fraction of the released proteins. Further protein analysis was done by SDS-PAGE (12% v/v).

### 2.4. Optimization of BglA Expression

The optimization of BglA was carried out using three media, namely, LB, Terrific broth (TB), and M9NG [13]. For each medium, culturing was initiated by refreshing an overnight culture in 50 ml to 0.8 OD_600_ and then induced. Each medium was investigated for a decline phase time span by inducing with either IPTG or lactose (autoinduction). After determining the time frame for each medium, different inducer concentrations were tested. Tested final concentrations for IPTG were 0.1, 0.3, 0.5, 0.7, and 0.9 mM, while for lactose, 5, 10, 15, 20, and 25 mM were used. SDS-PAGE (12%) was performed for all optimization experiments as mentioned above. The gels were visualized using the Gel Doc XR System (Bio-Rad Laboratories, Hercules, CA, USA) and analyzed densitometrically for getting relative concentration or percentage (relative to quantified total cellular protein by Bradford assay) using Image Lab Software 6.0.1 (Bio-Rad Laboratories, Hercules, CA, USA).

### 2.5. Characterization of BglA

#### 2.5.1. His-Tag Purification of BglA

Purification of BglA enzyme was performed on the soluble fraction obtained after the lysis of induced *E. coli* BL21-CodonPlus (DE3)-RIPL cells. First, the soluble fraction was heat-treated at 65°C to precipitate the heat-sensitive *E. coli* proteins. The sample was centrifuged at 7000 RPM for 15 minutes, and the supernatant was processed for His-tagged column chromatography using Ni-NTA resin (Thermo Scientific) according to the manufacturer's protocol. The purified protein was first quantified using Bradford's method and spectrophotometry at 280 nm. The quantified protein was then analyzed by SDS-PAGE (12% v/v) for the approximate molecular weight.

#### 2.5.2. Enzymatic Activity

To find out the enzymatic activity of the purified BglA (salicinase), salicin (Sigma) was used as a substrate. Activity assays were done in 1.5 M Tris-Cl buffer (pH 7.0) with a final enzyme concentration of 10 ng. A series of different substrate concentrations ranging from 2.5 to 20 mM were incubated with enzyme at 50°C for 30 minutes in a total volume of 150 *μ*l. The liberated reducing sugars were quantified by 3,5-dinitrosalicylic acid (DNS) reagent. The incubated enzyme substrate mixture (150 *μ*l) was mixed with 150 *μ*l of DNS and boiled for 10 minutes to produce color. Final absorbance was taken at 545 nm [14] on a PR4100 microplate reader (Bio-Rad).

The BglA enzymatic activity was determined through the regression method by using the glucose standard curve. Unit of the activity was *μ*mol·min^−1^, which corresponds to one unit of enzyme (U). The Michaelis–Menten model of kinetics was applied on activity data to confirm graphical consistency prior to using it for the double-reciprocal Lineweaver–Burk plot. The plot gave *K*_m_ (Michaelis–Menten constant) and *V*_max_ (maximal reaction velocity) values for BglA.

#### 2.5.3. Temperature, pH Optima, and Stability

The optimum temperature for reaction was determined by incubating the separate reactions on a range of temperature (30–75°C) for 30 minutes in 1.5 M Tris-Cl buffer (pH 7). Similarly, for an optimum pH value of BglA reaction, a range of different pH values (pH 4–10) was used in separate reactions. Citrate-phosphate buffer (0.15 M) was used for pH 4–8, while 1.5 M Tris-Cl buffer was used for pH 8–10. Highest enzymatic activity in U/mg gave the optimum temperature and pH values for BglA.

The stability of the BglA against temperature was measured by incubating the enzyme for one hour at 25–75°C. Temperature interval was kept at 5°C. The stability against pH was determined by incubating the enzyme at ambient temperature for 30 minutes in 0.15 M citrate-phosphate buffer of pH 4–8 and 1.5 M Tris-Cl buffer of pH 8–10. The residual activities in U/mg gave the temperature and pH stability cutoff values for BglA.

#### 2.5.4. Circular Dichroism Analysis

Native structure of the recombinant BglA was confirmed by circular dichroism (CD) spectroscopy. Data were collected on a Chirascan-Plus spectrophotometer (Applied Photophysics), furnished with Peltier thermal-controlled cuvette holder. Protein solution containing 125 *μ*g/ml in 20 mM Tris-Cl pH (8.0) was scanned over a wavelength range of 186–280 nm at 20°C in duplicate manner using quartz cuvette of path length 1 mm at a bandwidth of 1 nm. Secondary structure content was estimated using CD spectrum deconvolution software (CDNN) [15].

## 3. Results and Discussion

### 3.1. Overexpression and Optimization of BglA in Different Media

**Figure 1:**
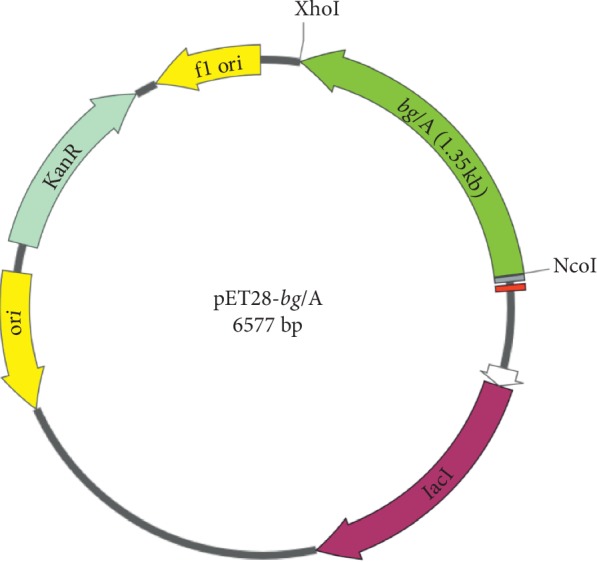
Graphical representation of pET28a-bglA. Expression vector was created by insertion of bglA gene between NcoI and XhoI sites. Inserted sequence was adjacent to lac operator and terminates at 6xHis-tag included in the frame. Kan^R^: kanamycin resistance gene, Ori: origin of replication. The sketch was created using SnapGene® software (GSL Biotech).

**Figure 2:**
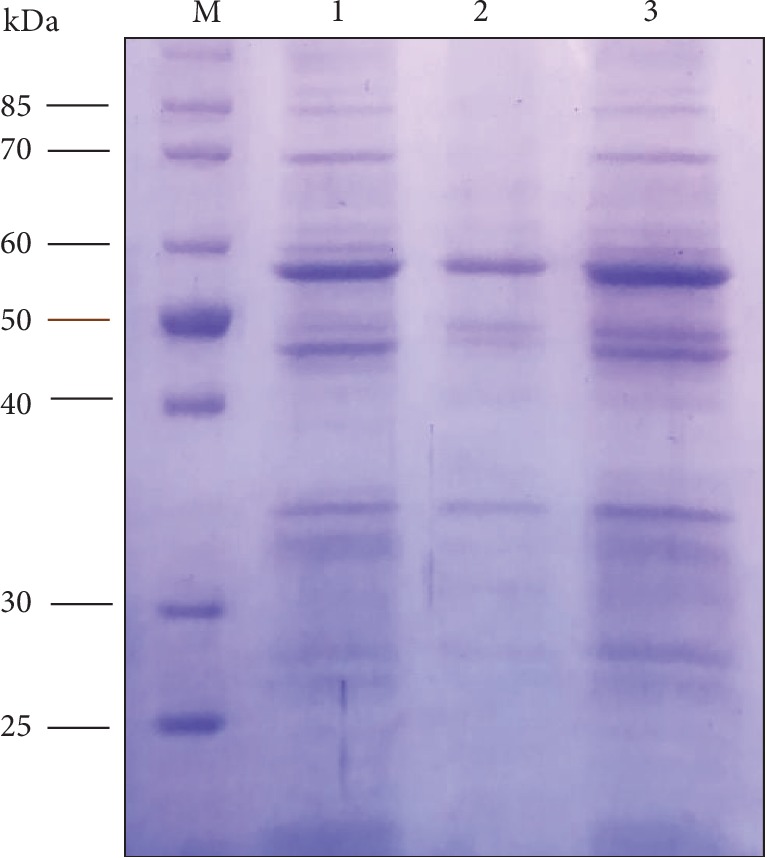
Expression analysis of BglA protein in *E. coli* BL21-CodonPlus (DE3)-RIPL by 12% SDS-PAGE. Lane M: protein standard marker; Lane 1: soluble proteins showing expression of BglA at ∼53 kDa; Lane 2: insoluble proteins; Lane 3: total cellular proteins showing expression of BglA at ∼53 kDa.

The *E. coli* BL21-CodonPlus (DE3)-RIPL cells harboring pET28a-bglA vector (Figure 1) confirmed the expression of BglA protein after being induced with IPTG. Postexpression protein analysis by 12% SDS-PAGE showed that the enzyme was mainly expressed as a soluble active protein having an approximate molecular mass of 53 kDa which coincides with the calculated mass (Figure 2).

To obtain the maximum expression of BglA in different media, a maximum cell density (OD_600_) of 3.6 post 6 hours of induction with IPTG with an expression level of ∼50% was achieved in LB, while autoinduction with lactose in LB medium gave a maximum cell density (OD_600_) of 6.5 though maintaining an expression level of ∼50%, whereas a maximum cell density of 5.5 and 16.4 in TB medium was achieved in 8 hours and 16 hours when induced with IPTG and lactose, respectively, with an expression level of ∼70%. However, maximum cell density in autoinduction medium M9NG was 13.4 with an expression level of ∼70%. M9NG was also induced with IPTG with a maximum OD_600_ of 3.47 and an expression level of less than ∼40%. A comparison of all these outcomes in three media is given in Table 2. BglA from *C. thermocellum* was first purified in 1979 [16] at ∼50 kDa and was reported through DISK electrophoresis. Another study elucidated the coding sequence of bglA gene and predicted its size to be ∼51.5 kDa [17]. The nucleotide sequence and size of cloned bglA in our study coincides with both of the prior studies. Several beta-glucosidases from other thermophilic bacteria have also been reported with similar sizes. Slight variations in size by different studies may be due to mass estimation by SDS-PAGE rather than by mass spectrometry [1, 4].

### 3.2. Solubility Analysis and Purification of BglA

**Figure 3:**
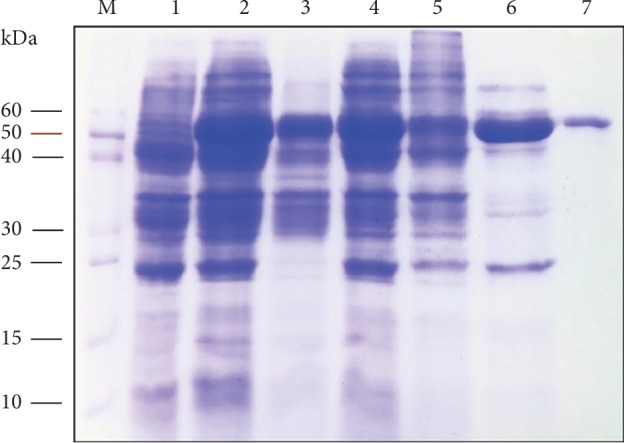
Solubility analysis and purification of BglA by 12% SDS-PAGE. Lane M: protein standard marker; Lane 1: control (pET28a, without gene of interest); Lane 2: pET28a-bglA, total cellular protein (TCP) of *E. coli* BL21-CodonPlus (DE3)-RIPL cells showing expression of BglA at ∼53 kDa; Lane 3: insoluble fraction of TCP; Lane 4: soluble fraction of TCP showing expression of BglA at ∼53 kDa; Lane 5: pelleted proteins after heat inactivation of soluble fraction; Lane 6: supernatant proteins after heat inactivation of soluble fraction; Lane 7: purified His-tagged BglA protein through Ni^2+^-NTA chromatography.

After media optimization, M9NG (1 liter culture) was utilized for large scale production, and 100 ml cell lysate yielded ∼75% expression of BglA protein relative to the total cellular protein (TCP). The soluble fraction of the lysate contained ∼75% soluble BglA of the total expression while ∼25% remained as insoluble (Figure 2). Heat inactivation of the soluble fraction precipitated out ∼20% of the protein leaving ∼30% biologically active BglA. This 30% was purified up to a purity level of ∼98% BglA by Ni-chromatography which resulted in a yield of ∼15% (Figure 3). Along with relative yield determination by densitometry after SDS-PAGE, protein content was also quantified at every purification step. The purified protein was quantified through Bradford assay which amounted to be ∼3.8 mg/ml. A summary of BglA activity, percentage recovery, and fold purification is shown in Table 3. Several studies have reported soluble expression and purification of BglA from different bacterial linages of *Thermotoga maritima* (*T. maritima*) and *Bacillus halodurans* (*B. halodurans*) using the *E. coli* expression system in the range of 0.5–1 mg/ml (∼70% of TCP). These studies used LB and M9NG as growth media, while purification methods like ammonium sulfate precipitation and metal affinity chromatography were used [4, 18].

### 3.3. Enzymatic Activity and Stability of BglA at Different Temperature and pH

**Figure 4:**
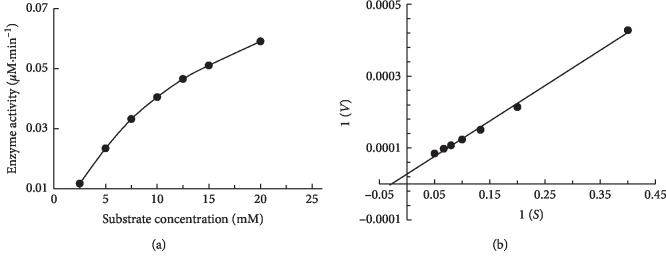
Enzymatic activity of BglA. (a) Lineweaver–Burk plot having substrate (salicin) concentration in mM on *X*-axis and enzyme (BglA) activity in *μ*M·min^−1^ on *Y*-axis; (b) reciprocated substrate concentration and enzyme activity values from which Michaelis–Menten constant was derived through nonlinear regression.

BglA had a maximum activity of 11.8 kU/mg with salicin. The hydrolysis kinetics of salicin substrate by beta-glucosidase determined for 2.5 to 20 mM concentration range are plotted (Figures 4(a) and 4(b)). The calculated values for *K*_m_ and *V*_max_ were 19.83 mM and 0.12 *μ*mol·min^−1^, respectively, for salicin. The enzymatic activity of the BglA from native *C. thermocellum* has not been reported yet to our knowledge. BglA activities (*K*_m_) in other bacterial lineages like *B. halodurans* and *T. petrophila* have been stated to be 4 mM and 2.8 mM, respectively [1, 20].

**Figure 5:**
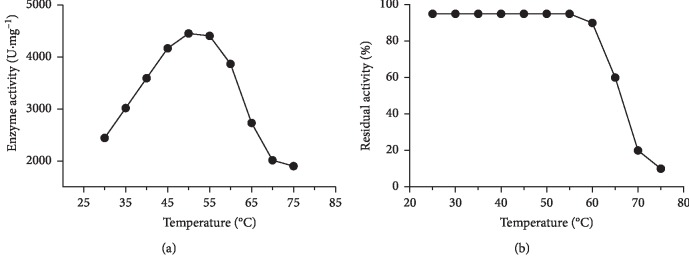
Optimum temperature characteristics and stability against temperature of BglA. (a) Enzyme activity of BglA (●) plotted against different temperatures (30–75°C). Maximum activity can be noticed at an optimum temperature, i.e., 50°C. (b) residual activity percentage of BglA (●) is plotted after incubation at different temperatures irrelatively and a sharp decrease in activity can be seen after 60°C.

BglA was analyzed for the optimum activity within a range of temperature, i.e., 30–75°C, and the enzyme showed maximum activity at 50°C (Figure 5(a)). The enzyme stayed active up till a temperature of 60°C after which there was a gradual decrease in its residual activity (Figure 5(b)).

**Figure 6:**
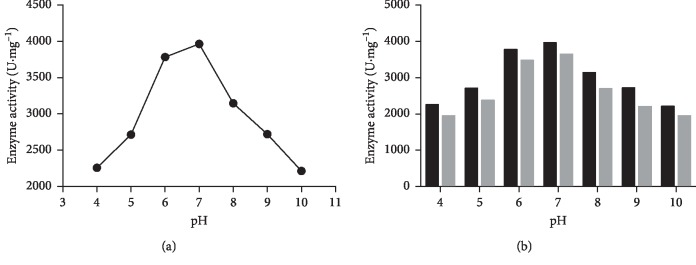
Optimum pH characteristics and stability against pH of BglA. (a) Enzyme activity of BglA (●) plotted against different pH (4–10). Maximum activity can be noticed at an optimum pH, i.e., 7.0; (b) residual activity comparison as bar graph of BglA showing actual activity (black bars) at different pH values and residual activity (Grey bars) plotted after incubation at different pH values irrelatively and an extent of decrease in the activity at each pH value can be seen.

Similarly, optimum activity within a range of pH (4–10) was analyzed for BglA, and it showed maximum activity at pH 7.0 and was considered to be optimum (Figure 6(a)). Moreover, the stability against pH was also seen up to pH 7.0, after which there was a sharp decrease in the activity (Figure 6(b)). The temperature and pH optima were in accordance with thermophilic nature of BglA's source. The other beta-glucosidase (BglB) of *C. thermocellum* cloned [3] had an optimum temperature of 45°C and an optimum pH of 5.6 in contrast. However, in our case, BglA had a better stability, i.e., 60°C than BglB which was 45°C.

### 3.4. Circular Dichroism Spectrum of BglA

**Figure 7:**
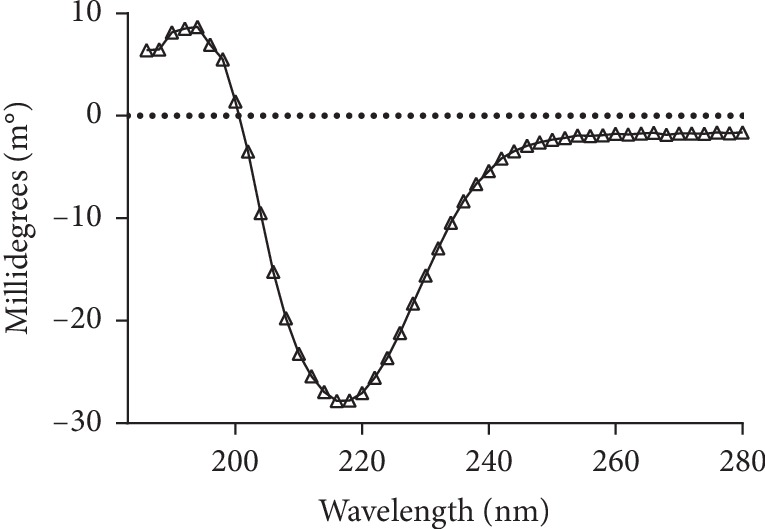
CD spectrum of BglA in 20 mM Tris-buffer (pH 7.0). The CD spectrum was measured from 186 to 280 nm in a 1 mm pathlength quartz cell with a step size and bandwidth of 1 nm each. The concentration of BglA was 100 *μ*g/ml.

CD spectrum of BglA is shown in Figure 7. A characteristic positive peak at 193 nm and a negative peak around 222 nm indicated a predominant *α*-helical content [21], whereas a positive peak near 195 nm and a negative peak at 208 nm showed presence of *β*-pleated sheets [22].

The secondary structure determined by CD spectroscopy in our study (as shown in Table 4) is comparable with the secondary structure content obtained, from both X-ray crystallography- (PDB ID: 5OGZ) and homology-based predicted models [23], along with in silico sequence-based prediction of secondary structure using Mufold [24]. The CD spectra revealed presence of alpha helix (43.50%) and a small percentage of beta sheets (10.60%).

**Figure 8:**
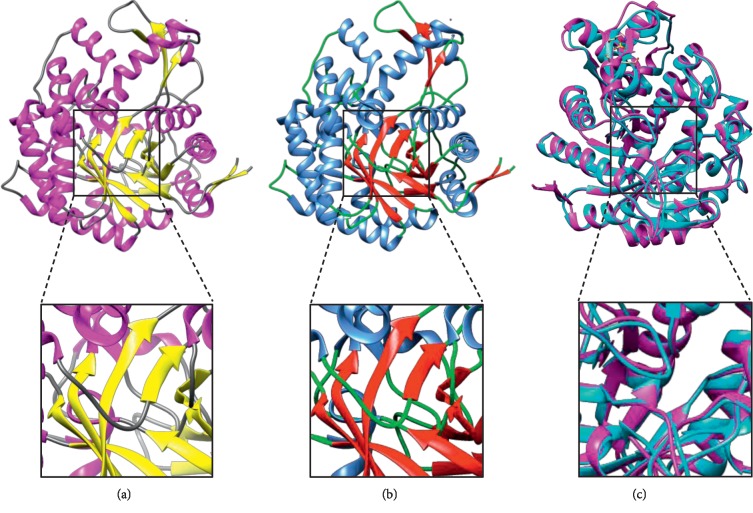
Tertiary structure model and comparison of BglA. (a) Modeled X-ray crystallographic structure of BglA (PDB ID: 5OGZ) showing alpha helices, beta sheets, and coils in magenta, yellow, and grey color, respectively; (b) homology-based predicted model of BglA showing alpha helices, beta sheets, and coils in cyan, red, and green color, respectively; (c) superimposed structure comparison of X-ray crystallographic and homology-based predicted models by using MatchMaker shown in magenta and cyan, respectively.

*In silico* rendering of the X-ray crystallographic model and homology-based predicted model was also constructed by using UCSF Chimera [25]. Visual elaboration of mainly present alpha helices and minor percentages of beta sheets can be seen in Figure 8. Pairwise sequence alignment was done by using MatchMaker command of Chimera followed by superimposing the structures according to those pairwise alignments.

## 4. Conclusions

The lysis activity of cellulose includes a chain of reaction, starting from endoglucanases and then beta-glucosidase, which ultimately converts cellooligosaccharides to glucose, but the product induces inhibition of prior enzymes. Our study aimed to clone and evaluate enzymatic activity and stability of BglA obtained from *C. thermocellum* in the *E. coli* expression system. The purified BglA showed soluble expression at ∼53 kDa with promising enzymatic activity (11.8 kU/mg) with salicin. The enzymatic activity and stability analysis of BglA also showed its thermophilic capabilities well above average. In conclusion, purified BglA is a suitable candidate for industrial utilization due to its high-level expression in *E. coli* with better enzymatic activity and thermostability.

## Figures and Tables

**Table 1:** Comparison of several microorganisms producing beta-glucosidase (BglA) and their activity in (U) with optimum pH and temperature.

Sr. No.	BglA-producing microorganism	Optimum temperature	Optimum pH	Enzyme substrate	Activity (U)	Reference
1	*Bacillus halodurans*	45	8.0	Lactose	95.0	[4]
2	*Bacillus licheniformis*	60	7.0	Glucose	45.0	[5]
3	*Penicillium oxalicum*	30	7.0	Cellulose	150.0	[6]
4	*Penicillium piceum*	70	4.0	Glucose	1.8	[7]
5	*Penicillium echinulatum*	50	4.8	Cellulose	1.5	[8]
6	*Penicillium* sp.	60	6.0	Cellulose	289.0	[9]
7	*Micrococcus antarcticus*	25	6.5	Cellobiose	0.058	[10]

**Table 2:** Summary of BglA optimization in different media (LB, TB, and M9NG) with IPTG and lactose as inducers.

Expression inducer	Growth medium	Max OD_600_	BglA % in TCP	BglA mg/ml${}^{\underset{-}{\text{a}}}$
IPTG	LB	3.69	50	0.6
	TB	5.49	70	0.7
	M9NG	3.47	40	0.5

Lactose	LB	6.56	50	1.5
	TB	16.46	70	2.0
	M9NG	15.18	70	2.2

*Note*. TCP: total cell protein. ${}^{\underset{-}{\text{a}}}$Concentration determined through relative densitometry.

**Table 3:** Summary of BglA recovery and activity at different stages of purification.

Purification stages	Total protein (mg/ml)	Percentage recovery (%)^a^	Percentage recovery (%)^b^	Total activity (U)	Specific activity (U/mg)	Fold purification
Crude cell extract	29.85	100	100	18704	627	1
Soluble fraction	17.84	60	70	14250	799	1.27
Treatment of soluble fraction at 65°C	15.57	52	30	13601	874	1.39
Ni-chromatography	3.8	13	15	11807	3107	4.96

^a^Percentage recovery (%) determined by Bradford assay [19]. ^b^Percentage recovery (%) determined by densitometry after SDS-PAGE.

**Table 4:** Secondary structure predictions of BglA by CD spectroscopy and comparison with other models.

Models	Alpha helix (%)	Beta sheets (%)
CD spectroscopy (BglA)	43.50	10.60
X-ray crystallography (PDB ID: 5OGZ)	43.63	15.57
Homology-based predicted model	42.11	14.86
Sequence-based prediction (Mufold)	43.90	13.80

## Data Availability

The data used to support the findings of this study are included within the article.
